# A Neural Mechanism for Background Information-Gated Learning Based on Axonal-Dendritic Overlaps

**DOI:** 10.1371/journal.pcbi.1004155

**Published:** 2015-03-13

**Authors:** Matteo Mainetti, Giorgio A. Ascoli

**Affiliations:** Krasnow Institute for Advanced Study, George Mason University, Fairfax, Virginia, United States of America; Hamburg University, GERMANY

## Abstract

Experiencing certain events triggers the acquisition of new memories. Although necessary, however, actual experience is not sufficient for memory formation. One-trial learning is also gated by knowledge of appropriate background information to make sense of the experienced occurrence. Strong neurobiological evidence suggests that long-term memory storage involves formation of new synapses. On the short time scale, this form of structural plasticity requires that the axon of the pre-synaptic neuron be physically proximal to the dendrite of the post-synaptic neuron. We surmise that such “axonal-dendritic overlap” (ADO) constitutes the neural correlate of background information-gated (BIG) learning. The hypothesis is based on a fundamental neuroanatomical constraint: an axon must pass close to the dendrites that are near other neurons it contacts. The topographic organization of the mammalian cortex ensures that nearby neurons encode related information. Using neural network simulations, we demonstrate that ADO is a suitable mechanism for BIG learning. We model knowledge as associations between terms, concepts or indivisible units of thought via directed graphs. The simplest instantiation encodes each concept by single neurons. Results are then generalized to cell assemblies. The proposed mechanism results in learning real associations better than spurious co-occurrences, providing definitive cognitive advantages.

## Author Summary

We introduce and evaluate a new biologically-motivated learning rule for neural networks. The proposed mechanism explains why it is easier to acquire knowledge when it relates to known background information than when it is completely novel. We posit that this “background information-gated” (BIG) learning emerges from the necessity of neuronal axons and dendrites to be adjacent to each other in order to establish new synapses. Such basic geometric requirement, which was explicitly recognized in Donald Hebb’s original formulation of synaptic plasticity, is not usually accounted for in neural network learning rules. More generally, the level of abstraction of current computational models is insufficient to capture the details of axonal and dendritic shape. Here we show that “axonal-dendritic overlap” (ADO) can be parsimoniously related to connectivity by assuming optimal neuronal placement to minimize axonal wiring. Incorporating this new relationship into classic connectionist learning algorithms, we show that networks trained in a given domain more easily acquire further knowledge in the same domain than in others. Surprisingly, the morphologically-motivated constraint on structural plasticity also endows neural nets with the powerful computational ability to discriminate real associations of events, like the sight of a lightning and the sound of the thunder, from spurious co-occurrences, such as between the thunder and the beetle that flew by during the storm. Thus, the selectivity of synaptic formation implied by the ADO requirement is shown to provide a fundamental cognitive advantage over classic artificial neural networks.

## Introduction

**Fig 1:**
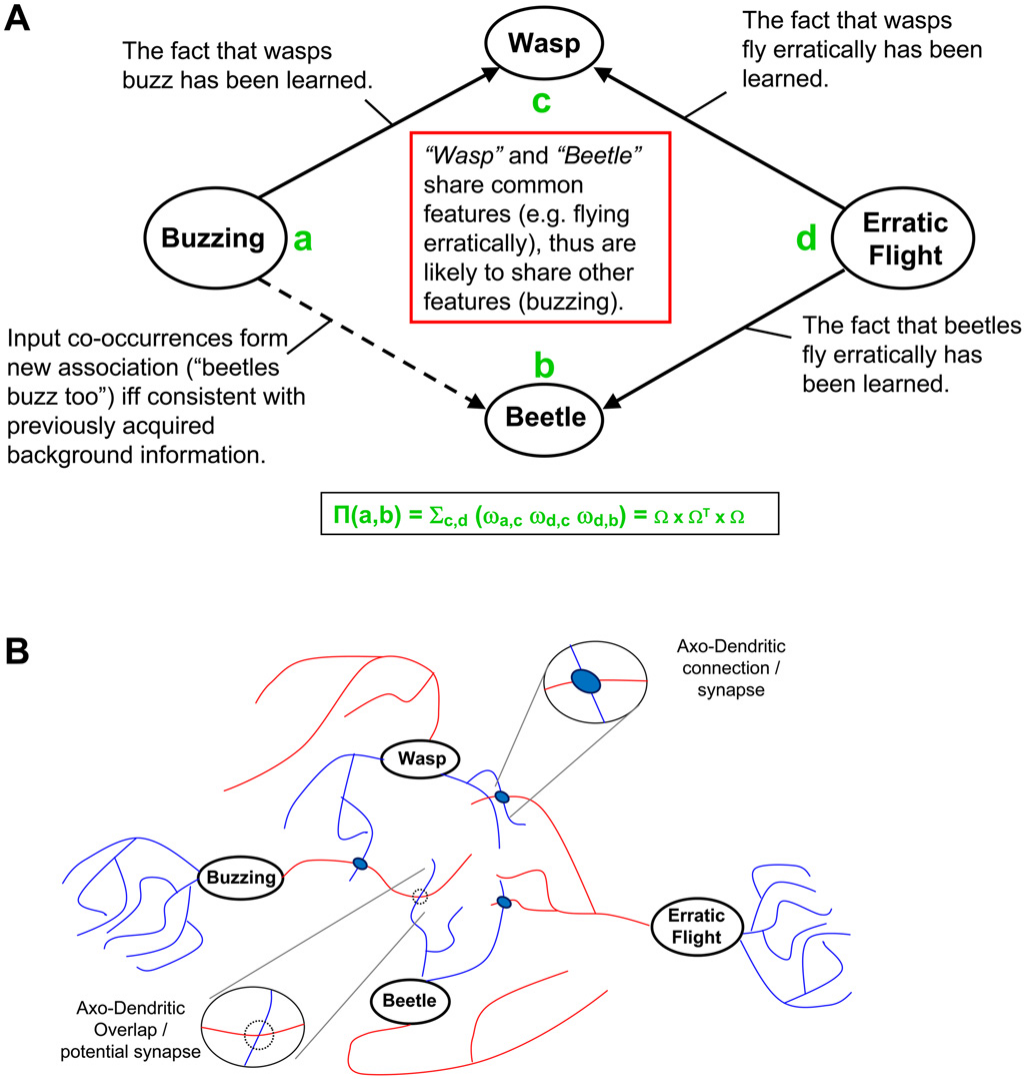
Instantiation of background information-gated (BIG) learning by the neuroanatomical mechanism of axonal-dendrite overlap (ADO). **A**. Cognitive model: Previously acquired background information, reflected in the structure of the association network, provides a gating mechanism for the formation of novel associations. The ability to acquire the new piece of information (associating the buzz to the beetle) depends on prior knowledge of relevant facts: in this example, that other buzzing animals (e.g. wasps) fly erratically. The green fonts *a*, *b*, *c*, and *d* refer to the *proximity* formula (also in green), fully described in the Materials and Methods. **B**. Neural correlate: In this simplified (“grandmother” cells) model, each concept of panel A is represented by a single neuron, with axonal and dendritic trees drawn respectively in red and blue. The axon of the “Buzzing” neuron has a synaptic contact with the dendrite of the “Wasp” neuron. Thus, it must pass close to the dendrites of other nearby neurons. Neurons are likely to be near each other if they receive synapses from the same axons. Here, “Beetle” is near “Wasp” as they both receive synapses from the axon of the “Erratic Flight” neuron. Thus, prior knowledge of relevant background information, instantiated by the three existing synapses, provides proper conditions to learn the new association, i.e. forming the “Buzzing”-“Beetle” synapse.

Reading about a newly discovered insect species, an entomologist can rapidly learn various details of their development, communication, and mating. Studying the same material, it is much harder for someone with different expertise to learn the same facts. While it is commonsense that new information is easier to memorize if it relates to prior knowledge, the cognitive and neural mechanisms underlying this familiar phenomenon are not established. More specifically, one-trial learning of “neutral” events, as opposed to emotionally charged or surprising experiences [1], is gated by knowledge of appropriate background information to make sense of the experienced occurrence [2, 3]. Consider experiencing for the first time the co-occurrence of a buzzing sound with the sight of a beetle (Fig. 1A). Learning that “beetles can buzz” may depend on background information that renders the “buzzing beetle” association sensible. Prior knowledge might include that wasps, flies, and bees also buzz. Such facts are *relevant* because they involve *related concepts*: these insects share several common associations with beetles (e.g. small size, crawling, flying, erratic trajectories). The remainder of this paper refers to this cognitive phenomenon as “background information gating” or BIG learning.

Mounting neurobiological evidence implicates formation of new synapses in long-term memory storage [4, 5, 6]. Building on those ideas, we propose a possible neuroanatomical correlate of BIG learning. The hypothesized mechanism is initially best illustrated under the over-simplifying assumption that associations are stored by connecting “grandmother” neurons, each corresponding to individual concepts (Fig. 1B). The computational simulations presented in this work, however, demonstrate that this same concept also seamlessly works with distributed neuronal representations.

In order to establish a synapse, according to Hebbian theory, the axon and dendrites of the two co-activated neurons must be juxtaposed [7]. We henceforth refer to this “potential synapse” configuration [8] as *axonal-dendritic overlap* or ADO. Intuitively, the reason the axon passes near the dendrite is because it is connected to other dendrites in that vicinity. Why then is the potential post-synaptic dendrite close to other dendrites contacted by the potential pre-synaptic axon? Wiring cost considerations suggest that neurons should be placed nearby if they receive synapses from the same axons [9]. If knowledge representation is stored in pairwise neural connections [10], this particular topology should correspond to relevant background information. Here we formulate this notion quantitatively with a new neural network learning rule, demonstrating by construction that ADO is a suitable mechanism for BIG learning.

In our model, neural activation reflects associations sampled from various graphs taken as a simplified representation of everyday experience. Specifically, every instant of experience is represented as a subset of co-occurring elementary observables, each corresponding to a node of a “reality graph,” in which edges denote probability of co-occurrence (see S1 Text 1.1 for a more extended description). We study networks pre-trained with an initial connectivity by comparing their ability to learn new information that is related or unrelated to prior knowledge. Such pre-existing background information may derive from repetition learning [11] or from experience earlier in life: if the BIG ADO were enforced from the start in a fully disconnected network, no new synapses could ever form. The simplest instantiation encodes each concept by single neurons; results are then shown to generalize robustly to realistic cell assemblies. Noticeably, the proposed mechanism results in learning real associations better than spurious co-occurrences, providing definitive cognitive advantages.

## Materials and Methods

The original simulation software used in this work was written in R, and the source code is freely available at http://krasnow1.gmu.edu/cn3/BigAdoAllCode.zip. Here we explain the research design pertaining to the findings reported in the main text. The detailed methodologies are more thoroughly described in S1 Text 2.1–2.4.

### Neural Network Model and the BIG ADO Learning Rule

This work assumes the classic model of neural networks as directed graphs in which nodes represent neurons and each directional edge represents a connection between the axon of the pre-synaptic neuron and the dendrite of the post-synaptic neuron. The network only contains excitatory neurons. In this model, formation of new binary connections (a form of *structural plasticity*) underlies associative learning, and knowledge is encoded by the connectivity of the network [10].

Activity-dependent plasticity is traditionally framed in terms of the Hebbian rule: “When an axon of cell *a* is near enough to excite cell *b* and repeatedly or persistently takes part in firing it, some growth process or metabolic change takes place in one or both cells such that *a*’s efficiency, as one of the cells firing *b*, is increased” [7]. Many variants of Hebbian synaptic modification exist [12], often summarized as ‘*neurons that fire together wire together*’. This popular quip, however, misses the essential requirement, clearly stressed in Hebb’s original formulation, that the axon of the pre-synaptic neuron must be sufficiently close to its post-synaptic target for plasticity to take place.

The learning rule introduced in this work implements a form of structural plasticity in neural networks that incorporates the constraint of proximity between pre- and post-synaptic partners or axonal-dendritic overlap (ADO): if two neurons *a* and *b* fire together, a connection from *a* to *b* is only formed if the axon of *a* comes within a threshold distance from a dendrite of *b*. In mathematical terms, this condition can be defined as a non-symmetric real-valued function between neurons corresponding to the distance from the axon of the candidate pre-synaptic neuron to the dendrite of the post-synaptic neuron.

Now we introduce an approximation to express the axonal-dendritic overlap between neurons in terms of the connectivity of the rest of the network on the basis of two assumptions. The first assumption is that the axon of *a* passes near the dendrite of neuron *b* because it connects to another neuron *c* that is near neuron *b*. This assumption corresponds to a principle of parsimony in the use of axonal wiring: since the goal of axons is to carry signals to other neurons, the locations of axonal branches are part of trajectories towards synaptic contacts. The second assumption is that if neurons *b* and *c* are near each other, it is because they are both contacted by the same set of axons, which we generically call *d* (Fig. 1). This assumption presumes optimal neuronal placement once again to minimize axonal wiring, consistent with the existence of topographic maps e.g. in the mammalian cortex [13], but also in invertebrate nervous systems [14].

These two assumptions can be combined into the assertion that the tendency of the axon of neuron *a* to overlap with a dendrite of neuron *b* increases with the number of neurons *c* and *d* such that *a* is connected to *c* and *d* is connected to both *b* and *c*. This idea is quantified by the following *proximity* (*π*) function:

$$\pi (a,b)\;=\Sigma_{c,d}(\omega_{a,c}\times \omega_{d,c}\times \omega_{d,b}),$$

where *ω*_*a*,*c*_ equals 1 if and only if the axon of *a* connects to the dendrite of *c*, and 0 otherwise (likewise for *ω*_*d*,*c*_ and *ω*_*d*,*b*_), and the indices *c* and *d* run over all neurons in the network (see also Fig. 1A). The above formula can be elegantly expressed as the product of three matrices:

$$\prod =\Omega Ω\times Ω\Omega^{t}\times Ω\Omega ,$$

where *Ω* = {*ω*_*m*,*n*_} is the (binary) network connectivity (also called adjacency matrix), with the number of rows and columns equal to the number of neurons in the network, and each row and column representing a neuron’s pre- and post-synaptic contacts, respectively, with all other neurons; *Ω* ^*t*^ is the transpose matrix in which every row is substituted with the corresponding column and vice versa (this operation is equivalent to switching axons and dendrites for each neuron); and *Π* = {*π (m*,*n*)} is the proximity matrix, which (like *Ω*) is square and non-symmetric.

The results presented in the main text are obtained by choosing a value for the proximity threshold *θ* in order to discriminate between proximal and distant pairs of neurons: *a* is deemed proximal to *b*, that is there is a potential synapse between *a* and *b*, whenever *π* (a,b) > *θ*. The proximity threshold is one of several parameters that have to be fixed when running simulations of an actual system; robustness of the mechanism is discussed in S1 Text 3.2. As an alternative to such a discontinuous threshold, we also implemented a probabilistic criterion for relating potential connectivity to proximity. In this case, the probability of *a* and *b* being proximal was not a binary function of proximity but it instead followed a sigmoid curve. This probabilistic variant, while introducing an additional source of noise in the simulations, yielded results (also described in S1 Text 3.2) that confirmed the main results of this work. However, this more general approach also increases the complexity of the model, by requiring the specification of an additional parameter to define the slope of the sigmoid.

Note, in a similar vein, that the above proximity formula seamlessly extends to non-binary connectivity matrices. For instance, network connectivity could be expressed as a matrix *Ω* recording not just the existence of a connection between two neurons, but the number of their physical contacts or other relevant measures, such as the stability of the synapses [15]. In the simple formulation used in this work, which presumes optimal neuronal placement to minimize axonal wiring, high proximity values make axonal-dendritic overlap likely, but not absolutely warranted.

The learning rule described above relates closely to earlier works proposing similar learning mechanisms to explain generalization and grammatical rule extraction. Most strikingly, a learning procedure with a very similar structure was described [16] to explain a generalization of a novel sequence (b-d) based on experienced sequences (a-c), (a-d), and (b-c). Despite this similarity (which we discovered during peer-review), the formulation introduced in the current work was derived independently, starting from the interpretation in terms of axonal-dendritic overlaps and structural plasticity. More generally, circuit connectivity, synaptic plasticity, and neuronal placement are interrelated in a broad class of other common neural network approaches, including Kohonen-type self-organizing maps [17]. In our model, the ADO constraint on structural plasticity is reduced to simple topological proximity rather than physical distance between neurons. Moreover, the application to background information-gated learning, the neural network implementation, and the analyses presented here are all novel.

To explain why axonal-dendritic overlap (and the approximation captured by the above proximity formula) constitutes the neural correlate of background information gating (BIG), we revert to the (admittedly simplistic) “grandmother cell” interpretation in which each individual neuron represents a corresponding observable (Fig. 1B). With such a one-to-one mapping in place, existing synapses reflect learned associations between previously co-occurred observables (solid arrows in Fig. 1A), altogether constituting already acquired knowledge. When witnessing a new co-occurrence between the two observables *a* and *b*, the association of their internal representations will only be allowed if consistent with prior relevant knowledge, ultimately corresponding to background information.

### Pre-Training and Testing Design

This work investigates the computational characteristics of the BIG ADO learning rule starting from well-defined reality-generating graphs (described in the next sub-section of these Materials and Methods). In the general simulation design, the network of the agent’s internal representation is created by copying the set of nodes from the reality-generating graph, but connecting them by sampling only a subset of edges. This process produces a network effectively encoding a certain amount of knowledge of reality consistent with prior experience. The same result would be obtained by “pre-training” a(n initially) fully disconnected network with the common “firing together, wiring together” rule (without BIG ADO filter) and sequentially activating pairs of neurons corresponding to the sampled subset of the reality-generating graph.

This design models the agent’s representation of background information related to previously experienced aspects of reality. Such a set-up allows investigation of the effect of the BIG ADO filter on subsequent learning. In the testing phase, further experience is sampled from not-yet learned edges of the reality-generating graph. These can be chosen so as to represent co-occurrences of observables more or less closely related to the pre-trained knowledge (mimicking expert or novice agents, respectively). Specifically, when initially connecting the neural network, we select the pre-training subset of edges non-uniformly from the reality-generating graph, such that distinct groups of nodes are differentially represented. For example, if the neural network is pre-trained with 50% of the edges from the reality-generating graph, three quarters of these edges can be sampled from half of the nodes, and one quarter of the edges from the other half. The resulting neural network is an “expert” on half of the reality-generating graph (because it knows a majority of the corresponding structure), and a “novice” on the other half (where it only knows a minority of the structure). In the “learning test” phase, the network is presented with new edges selected either from within the domain of expertise (that is, from the one quarter of edges not used in pre-training) or from the outside (from the three quarters of unused edges in the other half of nodes). The network learns new edges only if the proximity of the corresponding nodes is above threshold.

Moreover, two (or more) edges of the reality-generating graph can be presented at once (e.g. x-y and w-z) to allow measurement of differential learning between the “real” and “spurious” associations. The former types reflect actual edges in the reality-generating graph (i.e. x-y and w-z), while the latter correspond to “random” co-occurrences (x-w, x-z, y-w, and y-z).

The requirement of axonal-dendritic overlap for the formation of new connections is implemented by ways of the proximity function, which itself depends on pre-acquired connectivity. Thus, if the BIG ADO filter were in place from the beginning, no synapses would ever form in the network. The above pre-training design, which circumvents this impasse, can be justified by a two-stage developmental model [18]. Early in development, neurons are still optimizing their placements, and axonal branches undergo frequent rearrangements; in the subsequent mature stage, experience-dependent synapse formation and pruning are still common, but neuronal wiring is much more stable. Nevertheless, the “pre-training” model adopted here is also consistent with non-developmental scenarios. Even in adulthood, growth processes can be triggered by continuous repetition or by neuromodulation reflecting emotionally salience (e.g. shock, pleasure, etc.). These conditions can explain the acquisition of prior knowledge (background information). The BIG ADO filter, in contrast, constitutes a neuroanatomically-inspired model of one-trial, emotionally neutral learning.

### Word Association Graph

**Fig 2:**
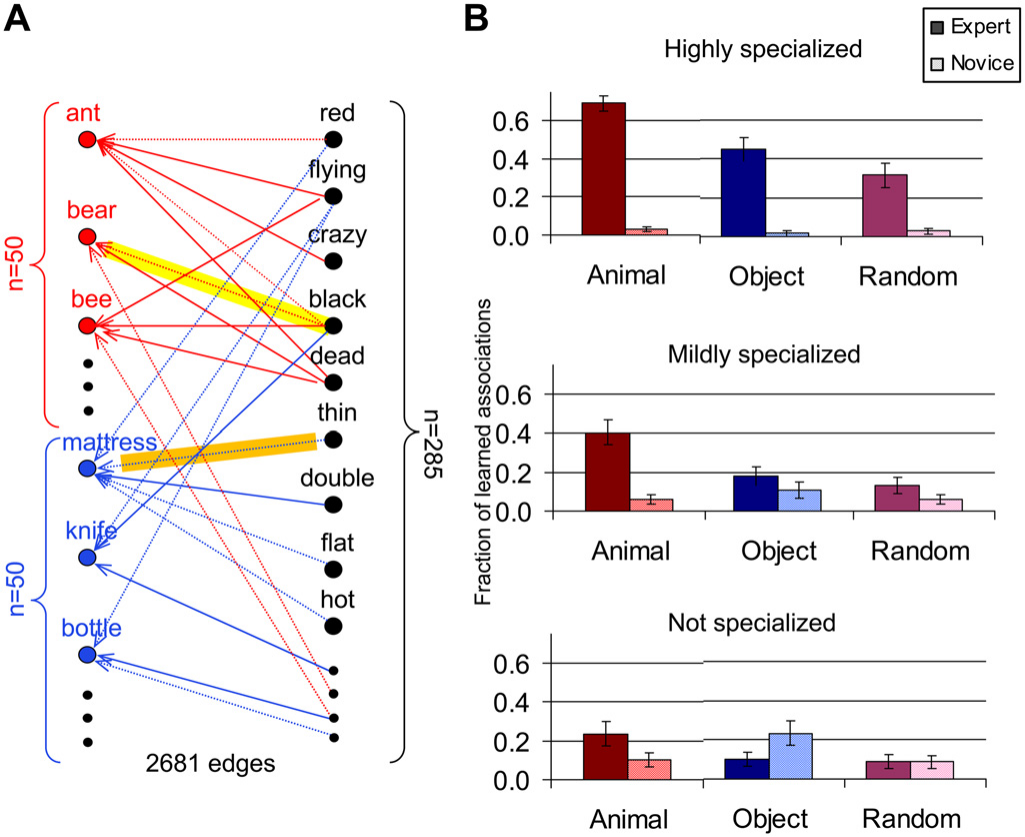
Word association with grandmother neurons. **A**. Adjective-noun associations in different domains of expertise: Portion of the bipartite association graph extracted from Wikipedia based on adjective pairing frequency for animals (red) and objects (blue) nouns. Arrows represent associations that have been learned during pre-training (solid lines) as well as those present in the bipartite graph but not used for pre-training (dotted lines). This example illustrates greater pre-training with animal associations (“animal expert”). Consequently, this network will be more likely to acquire newly presented associations that belong to the *animal* class (yellow highlight) as opposed to the *object* class (orange highlight). **B**. Background information-gated learning in the word graph: Proportion of newly acquired associations in the bipartite association graph. Networks were pre-trained with half of the edges, varying the amount of expertise from *highly specialized* (top row: 40% animal edges and 10% object edges or vice versa) to *mildly specialized* (middle: 30%-20% animal-object edges or vice versa) to *not specialized* (bottom: 25%-25%). A third network was pre-trained with the same proportions of two arbitrary subsets of edges in a random equivalent bipartite graph. The expert groups (left to right pairs in each row: animal, object, random) always outperformed the “novice” group (object, animal, random). The improved learning for animals relative to object (and random) cases is due to intrinsic background information (see text).

The dataset of word associations used in the first test of the BIG ADO learning rule (Fig. 2A-B) was derived from a compilation of noun/adjective pairings in Wikipedia. In its original form, it consisted of 32 million adjective-modified nouns (http://wiki.ims.uni-stuttgart.de/extern/WordGraph). After identifying nouns corresponding to animals and household objects, we skimmed infrequent adjectives and removed ambiguous terms (see S1 Text 2.1 for exact protocol). The resulting bipartite graph consisted of 50 animal nouns, 50 household object nouns, 285 adjectives and 2,682 edges (1,324 for animals and 1,358 for objects). Next, two networks were pre-trained by connecting half of the noun-adjective pairs from the graph. One of the networks associated more edges pertaining to animal nodes (becoming an animal expert and object novice), while the other associated more edges pertaining to object nodes (object expert, animal novice). Moreover, the amount of specialization was also varied to mimic different levels of specialization. This was achieved by varying the ratio between animals and objects learned in pre-training. Learning was then tested on the other half of the noun-adjective pairs using the BIG ADO rule with a proximity threshold (*θ* in equation 1) of 6. In the random equivalent graphs, edges between 100 “noun” nodes and 285 “adjective” nodes were generated stochastically by preserving both the overall noun and adjective degree distributions of the word graph. In this “control” condition, networks were pre-trained with expertise on one arbitrary subset of nodes.

The “intrinsic background information” of a noun class can be quantified from the bipartite graph with the *Proximity* function and Pearson’s product-moment correlation coefficients (S1 Text 3.1). Specifically, consider the proximities of a noun with the set of all adjectives: the correlation of these values can be then computed between any two nouns. The intrinsic background information of a noun class will be reflected by a statistically larger mean correlation coefficient over all pairs of nouns within that class than over all pairs of nouns from two different classes. The mean correlation was significantly greater for animal-animal than the animal-object pairs (0.69 *vs*. 0.47, p<10^-4^), while there was no statistical difference (p>0.1) between the mean correlations of the object-object (0.48) and object-animal (0.46) pairs (see S1 Text 3.1 for details).

### BIG Learning in Watts-Strogatz Networks

**Fig 3:**
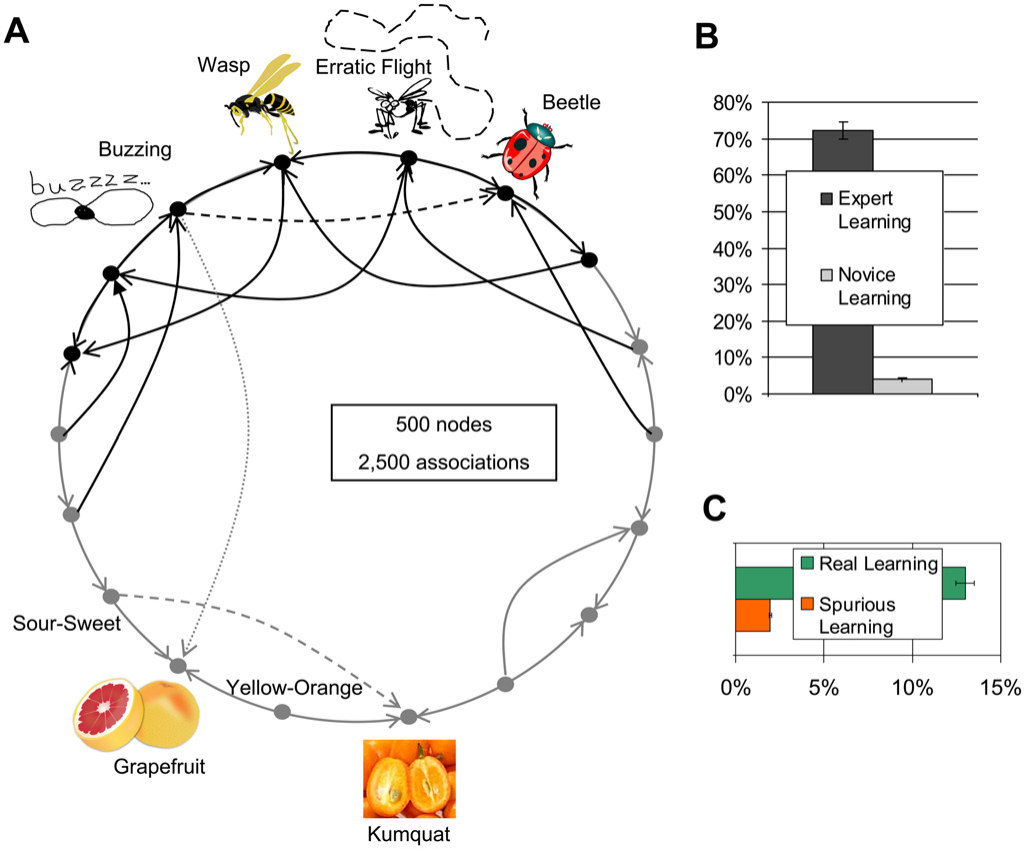
The cognitive value of BIG computations. **A**. BIG ADO in generic co-occurrence graphs: Simplified representation of the Watts-Strogatz graph-based model. During pre-training, half of the associations the network learns (solid lines) correspond to edges terminating in 20% of the nodes (black: “domain of expertise”). The other half is sampled from the remaining 80% of the graph (gray: novice domain). After pre-training, the ability to learn new (dashed) associations is tested both within and outside the domain of expertise. If two or more pairs of nodes are co-activated at once, spurious associations (dotted) could be learned across the pairs. **B**. BIG learning in small-world graphs: Differential ability of the pre-trained network to acquire new associations within (72.1±2.3%) and outside (3.9±0.4%) domain of expertise. **C**. Differentiating real from spurious associations: To discern the ability to learn real versus spurious associations in Watts-Strogatz graphs, pairs of new co-occurrences were presented, such as “buzzing beetle” and “buzzing grapefruit” (as if seeing/hearing a buzzing beetle while eating a grapefruit). The former is real (it belongs to the Watts-Strogatz graph), while the latter is spurious. Almost 13% of real associations were learned, including both those within and outside domain of expertise (black and gray lines in Fig. 3A), as opposed to less than 2% of spurious associations (dotted line in Fig. 3A).

To test the BIG ADO learning rule in more broadly applicable cases than noun-adjective associations, we generated small-world graphs adapting the algorithm of Watts and Strogatz [19]. Specifically, unless otherwise noted, Watts-Strogatz graphs were initially produced with degree 20 and 10% rewiring probability. Next, a random direction was selected for 90% of the edges, while the remaining 10% was made bidirectional. A random 20% of the nodes, along with all their incoming edges, were then labeled as belonging to the agent’s area of expertise. In the pre-training phase, networks were wired with a random set of edges of the graph, with the constraint that half of them must belong to the area of expertise, unless otherwise specified. The resulting connectivity consisted of a sub-graph of the initial graph, whose nodes in the area of expertise had higher average degree than those outside the agent’s expertise. In the “grandmother cell” implementation (Fig. 3), the BIG ADO threshold was set at 1. When the size of the graph (N) was varied to assess the robustness of the BIG ADO findings with respect to the parameter space, the degree (d) and the number of associations (edges) used to pre-train the network (T) also varied as d = N/50 and T = N×d/4, in order to keep the fraction of associations learned during pre-training constant.

### Extension of the ADO Rule to Cell Assemblies

**Fig 4:**
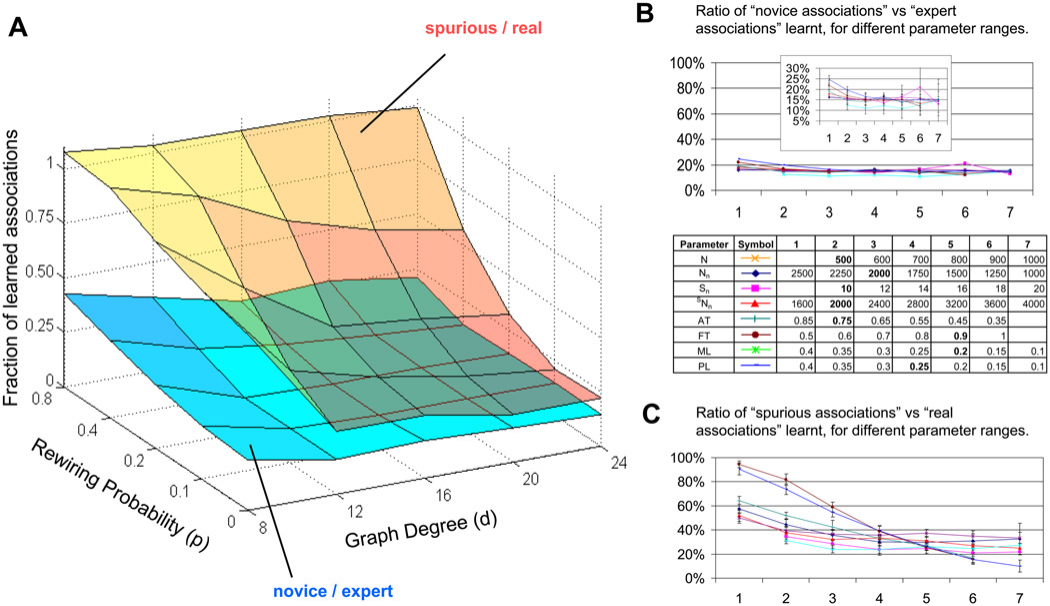
Generalization of ADO to biologically realistic mechanisms. **A**. BIG learning with cell-assemblies in small-world graphs of different connectivity: Ratios between the percentages of associations learned in the novice vs. expert domain (bottom surface) and for spurious vs. real co-occurrences (top surface) with varying graph degrees and rewiring probabilities when using cell assembly representation of Watts-Strogatz graphs. Lower rewiring probabilities and, to some extent, higher degrees improve the ability to discriminate real from spurious co-occurrences. These conditions correspond to highly clustered (as opposed to fully random) graphs. The ability to learn new associations within the domain of expertise remains more than double compared to a novice domain. **B**. Robustness of the BIG ADO mechanism: Ratios between the percentages of associations learned in the novice vs. expert domain with cell assembly representation of Watts-Strogatz graphs when varying (typically one at a time) several model parameters. The full ordinate scale is used to allow comparison with panel C, but the same data are also expanded in the **inset** to emphasize the invariance of the results (error bars: standard deviation). All parameter values are reported in the **table legend** below the plot (with default values in bold). The parameters and their abbreviations are: the number of nodes in the Watts-Strogatz graph (N), which also implies a change in the graph degree, d (kept at 2% of N) as well as the number of pre-training associations (corresponding to N×d/4, that is one half of the pool of available associations); the number of neurons in the network (N_n_) and the cell assembly size (S), whereas N was also varied together with S (^S^N_n_) so as to keep their ratio constant at 200; the activation threshold (AT), i.e. the fraction of neurons in the cell assembly that need to be synchronously active in order to “identify” the node of the graph represented by that assembly; the firing threshold (FT), i.e. the proportion of presynaptic neuron required to fire in order to activate a postsynaptic neuron; the matrix load (ML), i.e. the constant fraction of presynaptic neurons connected to each postsynaptic neuron in the cell assembly learning model; and the proximity load (PL), i.e. the (top) fraction of axonal-dendritic overlaps throughout the network that are considered to be potential synapses (see also S1 Text 2.4). **C**. Optimal conditions for one-trial learning of real but not spurious associations: Ratios between the percentages of associations learned for spurious vs. real co-occurrences with cell assembly representation of Watts-Strogatz graphs when varying the same model parameters as in Fig. 4B. The most tunable parameters are the firing threshold (neuronal excitability) and the proximity load (strength of BIG ADO filter: see S1 Text 2.4).

Neural network simulations with realistic cell assemblies (Fig. 4) implemented the Zip Net model [20], a computational enhancement of classic Associative Nets [21] that ensures optimal Bayesian learning [22]. Briefly, learning the association between two concepts A and B represented respectively by neurons a_1_, a_2_, …, a_s_ and b_1_, b_2_, …, b_s_, entails strengthening (or forming) synapses between co-active neurons and weakening or eliminating those between active and inactive neurons. Specifically, in the “incidence” matrix M with rows and columns respectively representing pre- and post-synaptic neurons, the entries in columns b_j_’s of all a_i_’s rows are increased while the remaining entries are decreasing by an appropriate amount to keep the total synaptic input constant (S1 Text 2.3).

In the pre-training phase, the connectivity matrix is generated from the incidence matrix simply by keeping a fixed number of synapses per neuron (those with highest weight), and setting the rest to zero. During BIG ADO testing, two neurons a and b can only form a new synapse upon co-activation if they have an axonal-dendritic overlap, which is expressed as the triple matrix product ΩΩ^t^Ω computed from the positive values of the incidence matrix (S1 Text 2.4). Lastly, retrieval works as a classic dendritic sum: given a stimulus A’ represented by neurons a’_1_, a’_2_, …, a’_s_, all the entries in the rows corresponding to the a_i_’s are added up for each column, and those sums exceeding a given firing threshold correspond to activated (post-synaptic) neurons. If enough neurons belonging to the same cell assembly B’ fire, concept B’ gets activated.

## Results

### Prior Knowledge Gates Learning of Word Associations by Grandmother Neurons

We tested the BIG ADO paradigm on a bipartite association graph derived from a compilation of 32 million noun/adjective co-occurrences in Wikipedia. We identified two classes of nouns (animals and household objects) and pre-trained two networks to learn a subset of the noun/adjective associations, each with “expertise” mostly in one of the two noun classes (Fig. 2A). Specifically, one network was pre-trained with a greater proportion of animal/adjective associations than of object/adjective associations (and vice versa for the other network). BIG learning facilitated networks to acquire new information that was related to the information already stored. Moreover, the magnitude of this phenomenon increased with the level of specialization between animals and objects (Fig. 2B). Note that, even in their “novice” domain of knowledge, networks cannot be completely “naïve.” Even if the pre-trained proportion of “novice” edges is lower than in the domain of expertise, it must still be non-zero or else no subsequent associations could be learned.

Interestingly, the effect was greater for animal expertise than for object expertise. Furthermore, more animal associations were learned when the network was pre-trained with the same number of animal and object edges. Both of these differences can be explained by two independent forms of background information: one intrinsic in the source data, and another dependent on the sample used to pre-train the network. The former was eliminated by repeating the simulations on random equivalent graphs (Fig. 2B: right bar pairs). Direct analysis of Pearson’s coefficients of the bipartite graph Proximity function (see Materials and Methods) confirmed that the noun/adjective association is more specific for animals than for objects (0.69 *vs*. 0.48, p<10^-4^).

### BIG Learning in Small-World Graphs: Ability to Differentiate Real from Spurious Associations

To validate the above results against broadly applicable cases besides word associations, we tested the BIG ADO learning rule in a general class of random small-world graphs [19] resembling real-world architectures, organizations, and interactions (Fig. 3A). Networks were pre-trained with samples of associations biased towards an arbitrary subset of nodes. As in the bipartite graph, the ADO filter gated subsequent learning of new associations by favoring those pertaining to this background information (Fig. 3B). Next we investigated the ability of BIG to differentiate between “real” and “spurious” associations. Most co-occurrences experienced in everyday life do not reflect real associations, but rather events that happened together by chance. For example, suppose you were eating a grapefruit while experiencing the buzzing beetle described in the *Introduction*. Why should buzzing be associated with beetle and not with grapefruit?

Hebbian models form both associations, relying on later experience to reinforce those that reoccur and eliminating the others [12], e.g. upon repeatedly dissociated experiences of eating a grapefruit without buzz and vice versa. Strikingly, the BIG ADO filter distinguished real from spurious associations (Fig. 3C), facilitating the ability to learn relevant co-occurrences over “occasional” ones the first time around. In a simple protocol, each experience consisted of the co-activation of two independent pairs of connected nodes in the Watts-Strogatz graph. The resulting six co-occurrences correspond to two real associations (between the two connected nodes in each of the pair) and four spurious associations (between neurons across the pairs).

Inspection of the simulation outcomes confirmed that spurious “buzzing grapefruit” co-occurrences were not remembered *because* they lacked relevant background information. In the pre-trained network, the axon of buzzing overlaps with the dendrite of beetle (high ADO) thanks to the already acquired buzzing-wasp, flying erratically-wasp, and flying erratically-beetle associations. Thus, the potential association buzzing-beetle ‘passes’ the BIG ADO filter. In contrast, buzzing and grapefruit have little if any axonal-dendritic overlap; thus, the corresponding association is not formed according to the BIG ADO mechanism. The learning differentials of both expert-over-novice networks and real-over-spurious associations increased with the bias towards a subset of nodes in the Watts-Strogatz graph, and were observed over a broad range of model parameters (see S1 Text 3.2 for additional results).

### Generalization to Realistic Cell Assemblies

The notion of representing mental states or elementary concepts in single (“grandmother”) neurons is appealing [23] but unrealistic [24]. Theories and experiments estimate that at least 50–200 cells take part in encoding each unit of thought [25, 26, 27]. Cell assemblies provide for redundancy, error-correction, and larger storage capacity. We thus extended the BIG ADO paradigm to cell assemblies. In cell assembly models, acquiring a new association between two co-occurring events entails formation of new synapses between the neurons representing one event and the neurons representing the other event. With the BIG ADO filter, forming synapse between a pair of co-active neurons requires appropriate pre-existing connections similarly to Fig. 1B, with the notable difference that the same neuron typically belongs to several cell assemblies.

Among the first (and simplest) neural network models employing cell assemblies are Willshaw’s Associative Nets [21]. Simulations with the Willshaw model confirmed the BIG ADO results with the word association graph (see S1 Text 2.3 for implementation detail and S1 Text 3.2 for analysis). However, the original Associative Nets achieve maximal storage capacity when cell assembly size is log-proportional to the number of neurons [20]. Such limitation on cell assembly size makes this approach unsuitable for learning realistic Watts-Strogatz graphs. A more sophisticated variant of this model, which achieves optimal Bayesian learning [22], attains excellent performance for cell assembly sizes compatible with those estimated for real brains. This latter model (Zip Nets) enabled cell assembly implementation of the BIG ADO mechanism with generic Watts-Strogatz graphs. In a typical configuration, the network learned 50% of novel associations within its domain of expertise, but only 9% unrelated to prior knowledge. When two node pairs (sampled randomly within and outside domain of expertise) were co-activated at once, 30% of real associations were learned vs. 7% of the spurious ones. Sampling only within or outside the domain of expertise, the learning proportions for real and spurious pairs were 50% and 12% or 9% and 3%, respectively.

Similar outcomes were consistently observed across a broad range of connectivity parameters in the small-world graphs. In particular, a substantially higher proportion of associations were learned within the domain of expertise than outside for any graph degree *d* (the average number of edges per node) from 8 to 24 and rewiring probability up to 80% (Fig. 4A). The rewiring probability R defines by construction Watts-Strogatz graphs as hybrids between regular (R = 0%) and random graphs (R = 100%). The fraction of spurious associations learned was substantially lower than that of real associations for degrees above 5 and rewiring probability below 50% (Fig. 4A). This suggests that prior connectivity (ADO) provides a biologically realistic neural correlate of background information and its ability to gate learning in any highly clustered networks. In clustered networks, two nodes are more likely to be interconnected if they are both connected to a third node. This is a common property of many types of graphs that extends beyond Watts-Strogatz networks [28].

### Robustness Analysis and Optimal Conditions

Although the adopted connectionist framework is an over-simplified model of nervous systems, this simplicity also reflects the foundational applicability of the BIG ADO learning rule. Specifically, the described mechanism does not depend on specific choices of parameters such as graph dimension, number of associations presented, learning threshold, and others. In particular, the main effect of axonal-dendritic overlap to selectively gate learning by background information was consistently reproduced in every combination of parameters conducive to adequate memory storage (Fig. 4B). Moreover, the discrimination between real and spurious associations with cell assemblies in small-world graphs was also largely unaffected by the choice of numerical values. Importantly, however, this latter effect varied quantitatively as a function of selected model parameters (Fig. 4C), such as the *proximity load*, which determines how topologically close an axon and a dendrite must be to constitute a potential synapse (see section 2.4 of S1 Text). This is the key parameter distinguishing BIG ADO from traditional Hebbian learning: a new synapse is formed between two neurons when they fire together *if and only if* a potential synapse is already present. Thus, certain circuits might be better designed than others to support efficient one-trial learning depending on their specific plasticity and excitability (see S1 Text 3.2 for additional results).

## Discussion

This report introduced a new biologically-motivated learning rule for neural networks that explains why it is easier to acquire knowledge when it relates to known background information than when it is completely novel [11]. The key idea is that this “background information-gated” (BIG) learning emerges from the necessity of neuronal axons and dendrites to be adjacent to each other in order to establish new synapses. Such basic geometric requirement was explicitly recognized in Hebb’s original formulation of synaptic plasticity, yet is not usually accounted for in neural network learning rules. The claim that existing structure matters for learning is not new [29]. However, the level of abstraction of current computational models of brain function fails to capture the details of axonal and dendritic shape.

The critical breakthrough of this work consisted of parsimoniously relating “axonal-dendritic overlap” (ADO) to circuit connectivity by assuming optimal neuronal placement to minimize axonal wiring. This corresponds to a fundamental neuroanatomical constraint: an axon must pass close to the dendrites that are *near other neurons it contacts*. The topographic organization of the mammalian cortex ensures that nearby neurons on average encode related information [30]. Incorporating this new relationship into classic connectionist learning algorithms, we found that networks trained in a given domain more easily acquire further knowledge in the same domain than in others. If the proximity threshold is set to zero, the model reverts to a traditional neural network unconditionally learning all associations. From this perspective, the BIG ADO rule could be considered as a biological constraint on learning.

However, to our initial surprise, the morphologically-motivated constraint on structural plasticity also endows neural nets with the powerful computational ability to discriminate real associations of events, like the sight of a lightning and the sound of the thunder, from spurious co-occurrences, such as between the thunder and the beetle that flew by during the storm. Thus, we surmise that the selectivity of synaptic formation implied by the ADO requirement provides a fundamental cognitive advantage over the unconstrained “fire together, wire together” plasticity rule of classic artificial neural networks. Of course the ability to associate completely unrelated facts or events may also be useful in many circumstances. Several different models have proposed that the hippocampus might be specialized for precisely that function, possibly leveraging its superior plasticity rate [31] or adult neurogenesis [32]. Our model suggests that this ability might also derive from the lack of topographic mapping in this structure (e.g. hippocampal area CA3). Moreover, the profuse axonal arbors of cortical neurons may enable access to a surprisingly large pool of intertwining dendrites through neurite outgrowth [33], perhaps providing a counter-mechanism to balance the BIG ADO rule.

The computational advantage of the BIG ADO algorithm over alternative learning rules can be quantified in terms of discrimination between real associations and spurious co-occurrences. If *k* pairs of real associations (A_1_-B_1_, A_2_-B_2_, …, A_k_-B_k_) are presented at the same time, BIG ADO selectively learns the correctly paired events over spuriously co-occurring ones (e.g. A_1_-B_2_, A_2_-B_1_, etc.). A “fire-together, wire-together” rule without ADO constraint can achieve similar selectivity by repetition. In this case, each association must be presented multiple times in order to attain the same discrimination power displayed by BIG ADO in one-trial learning. The number of required repetitions grows with the number *k* of real associations presented together and also depends on the structure of the association graph. For example, in the conditions of Fig. 3, BIG ADO learns real associations at a rate of 6:1 relative to spurious co-occurrences upon the first presentation. To obtain the same ratio in the absence of ADO if just five pairs are presented together, *each* association has to be repeated on average four times.

Mammalian brains display greatest plasticity during development, but certain cortical regions remain plastic throughout adulthood [34, 35]. Our research design is consistent with an initial phase of maximal plasticity, followed by a ‘mature’ state of conditional plasticity. Specifically, during pre-training, all witnessed associations are learned. Clearly, the anatomical constraint of axonal-dendritic overlap holds in all phases of development. However, the more prominent neuronal and axonal movements in earlier developmental stages would largely circumvent or alleviate the ADO filter. In practice, we pre-load the network directly with synaptic connectivity equivalent to that resulting from such an initial developmental phase (representing ‘background knowledge’). Afterword, the model preferentially learns associations related to previously acquired information. The resulting mature network not only avoids associating the (most numerous) spurious co-occurrences, but is also optimally structured to learn the associations most relevant to the environment in which it developed. Besides providing clear evolutionary advantages, these key features could also be applied in artificial intelligence and search engines.

Background information gating explains the familiar ability to form stable memories based on single experiences (as opposed to repetition). This process is complementary to (and as fundamental as) other factors known to control learning, such as valence and novelty. The proposed mechanism of axonal-dendritic overlap, based on the elementary anatomical organization of neuronal circuits, is also independent of neuromodulatory pathways likely to underlie alternative or parallel regulation of one-trial learning. This framework can also be useful to describe how semantic knowledge can be incorporated into existing knowledge. Moreover, the model offers a possible neural network correlate for the rapid memory consolidation occurring when new information is assimilated into a pre-existing associative “schema” or mental representation [36]. Other recent models have been proposed to explain the dependence of learning on prior knowledge [37].

The proposed BIG ADO learning rule is only conceptually related to axonal-dendritic overlap, as the anatomical data necessary to generate a complete model of all axons and dendrites in a network is still unavailable (see e.g. [38]). Realistically, potential synapses might work in synergy with additional mechanisms conducive to the same learning rule. For example, presentation of individual elemental associations (buzzing wasp, flying wasp, and flying beetle) may lead to the formation of cell assemblies representing associations between higher-order concepts and their properties (“flying insect”), as previously hypothesized [39], possibly supported by ongoing structural plasticity [40]. Moreover, axonal-dendritic overlap may provide powerful constraints for the recruitment of individual neurons into cell assemblies. While cell assembly selection has been proposed as the core of knowledge representation in neural systems [41], the underlying anatomical mechanisms have so far remained elusive [26]. Thus, the proposed link between neuronal structure and function may constitute an essential foundation for brain-based theories of cognition.

## Supporting Information

S1 Text Much ADO About BIG Learning: Supplementary Information. The single Supporting Information file (S1 Text) describing the model’s underlying assumptions, detailed methodologies, and supplementary results includes additional text, illustration, and references.
(DOCX)
