# Supplementary material for: A Neural Mechanism for Background Information-Gated Learning Based on Axonal-Dendritic Overlaps
Source: PLoS Comput Biol. 2015 Mar 13;11(3):e1004155. doi: 10.1371/journal.pcbi.1004155 (PMC4359104; doi:10.1371/journal.pcbi.1004155)
Supplement: S1 Text — The single Supporting Information file (S1 Text) describing the model’s underlying assumptions, detailed methodologies, and supplementary results includes additional text, illustration, and references. (DOCX) [file pcbi.1004155.s001.docx]

**Much ADO About BIG Learning: Supplementary Information**

This Supplementary Information includes three parts. The first part describes the computational assumptions underlying our approach. The second part provides extensive methodological details for all the studies presented in this work. The third part reports additional results that complement and expand on those illustrated in the main text.

**Part 1: Underlying Assumptions**

1.1: A model of knowledge

**Part 2: Detailed Methodologies**

2.1: Noun-adjective co-occurrence graph

2.2: Watts-Strogatz networks

2.3: Associative Nets and optimal Bayesian learning models

2.4: BIG ADO implementation with cell assemblies

**Part 3: Additional Results**

3.1: Word association: graph analysis, weights, and cell assemblies

3.2: From ADO to synapses: variations and robustness**Part 1: Underlying Assumptions**

**1.1: A model of knowledge**

Learning may be viewed as a process involving one or more *agents* (e.g. human beings) interacting with an objective reality in which they are embedded. This works assumes that certain aspects of reality relevant to learning can be represented as collections of observables. Each observable can be described with specific physical characteristics, such as a sound occurring in a given location and time with particular frequency and duration. A very large number of observables occur in every moment of time. A form of associative learning consists of an agent’s ability to store the information that two or more observables co-occurred.

Observables often occur together within a given time span because they are inter-related, such as a buzzing sound and a flying beetle. In most cases, however, the co-occurrence of two observables is the product of independent events. For example, many observables co-occurred the last time a beetle buzzed over the Leaning Tower of Pisa, including an airplane flying above, a tourist eating a grapefruit in the square below, and a dog chasing a cat. Only the flying beetle, however, was linked to the buzz, whereas all other events happened together by random coincidence^[[1]](#endnote-1)^. While independent observables can be assumed to be *completely* independent from each other, related observable are usually related to varying extents. For example, flying beetles sometime buzz, but not always.

These kinds of scenarios can be modeled by assuming that observable reality is generated from a *directed weighted graph*, whose nodes represent observables (each with a probability of occurrence) and edges represent probabilities of co-occurrence beyond chance (conditional probabilities). This “reality-generating” graph is supposedly very vast and only sparsely connected. In this framework, any moment of observable reality (that is, the collection of co-occurring observables at that moment) can be viewed as if sampled from the reality-generating graph.

Every agent embedded in reality only witnesses a subset of co-occurring observables depending on its individual perspective (physical location, state of attention, etc.). Thus, two agents embedded in the same reality will generally witness different collections of co-occurring observables. We assume that agents have the means to represent observables internally, and can learn to associate these internal representations from witnessing co-occurrences. The agent’s knowledge of reality can thus be also described as a graph, whose nodes correspond to represented observables and whose edges quantify the belief of a link between observables.

The above models of reality and knowledge are clearly over-simplified vignettes of much more complex phenomena. At the same time, this framework captures some basic elements of observable reality, cognitive content, and neural substrates all in terms of common graph theory. This shared mathematical formalism allows quantitative exploration of the possible relations among observables, cognition, and neural representation using concrete examples, such as word co-occurrence in natural language, corresponding semantic knowledge, and activation of networks of neurons.

One popular example is constituted by word graphs, whereas nodes are terms of a text corpus, and edges (symmetric or otherwise) are defined according to specific criteria, such as co-occurrence in a sentence or paragraph, and can be weighted (for instance by co-occurrence frequency). In the word graph instance employed in the main text, and further described in section 2.1 of this Supplementary Information, two categories of terms, nouns and adjectives, are considered distinctly, therefore resulting in a bipartite graph. Word graphs can also be constructed using dictionaries, whereas nodes are terms in the dictionary, and a (directed, un-weighted) edge between two terms signifies that the former is used in the definition of the latter. The co-occurrence graph (given a corpus) and the definition graph (given a dictionary) are only two simple examples of the many models of reality-generating graph that can be built with words.

The notion of reality-generating graph, however, extends beyond words, as illustrated by the opening example of the main text, which assumes our “real” reality and people as agents. Here, the link between the flying beetle and its buzzing sound is reflected by an edge between the corresponding nodes in the reality-generating graph. This relation, however, does not manifest itself until a flying beetle happens to buzz, creating a co-occurrence in the observable reality. When this occurs, some people might be close enough to the event and paying attention to it, so as to witness the co-occurrence. While several of these witnesses had already learned the association prior to this experience, some may learn it this time, but others will not learn it yet. Thus, even when presented with the same co-occurrence, some agents learn it while others do not.

Although the ability to learn a new association depends on the number of times the co-occurrence is witnessed, it is also modulated by other aspects of experience, such as valence (when reward or punishment are part of the same collection of observables) and arousal (e.g. surprise or expectation). Another major but less readily recognized factor determining whether an agent learns a new association between observables upon witnessing their co-occurrence is prior relevant knowledge. In the buzzing beetle case, this background information could include that others insects (wasps, flies, etc.), which share common features with beetles (small size, crawling, flying, erratic trajectories), also buzz. In this work we hypothesize a neuroanatomical mechanism, expanded in the next section, to explain the above described process of “Background Information Gated” (BIG) learning.**Part 2: Detailed Methodologies**

All software described here was written in R and tested on a Samsung R519 Intel Pentium duo (3GB RAM, Windows XP). The source code and data sets used in this work are available upon request.

**2.1: Noun-adjective co-occurrence graph**

We derived the first model of reality-generating graph to test the BIG ADO learning rule from a compilation of noun and adjective pairs in Wikipedia. In its original form it consisted of 32 million instances of an adjective modifying a noun, such as “sweet dream” or “black tea” (http://wiki.ims.uni-stuttgart.de/extern/WordGraph). The choice of a word graph was guided by the desire to enable intuitive interpretation of the results. In order to facilitate the definition of easily recognizable domains of expertise, we pruned the initial collection to a selected sub-graph by identifying two classes of nouns, animals and household objects, and skimming infrequent adjectives. Specifically, the noun class “*animals”* was derived from the Wikipedia list of over 150 names (http://en.wikipedia.org/wiki/List_of_animal_names). The second noun class, “*household objects,*” initially consisted of 70 terms (such as *table, microwave, pillow, bathtub, shoe*) manually compiled from surveying the interior of one of the authors’ house.

The two classes were ranked by number of associated (distinct) adjectives. For the animal class, the three nouns on top of the ranking (*dog, cat, horse*) were removed from the list, together with other nouns with multiple meanings (i.e. *mouse, ram, seal, human, crane*). The top 50 remaining animal names were selected. As for house objects, the top 11 (*book, radio, computer,* etc.), and bottom 9 (*fridge, tablecloth, napkin*, etc.) ranking nouns were discarded so as to keep the numbers of associated adjectives of the remaining object nouns similar to those for animal nouns. There were a total of 2,863 adjectives modifying at least one animal, and 3,421 adjectives modifying at least one object. From this list of adjectives we discarded those modifying 35 or more animals as well as 35 or more objects. Knowing that a given adjective is an attribute of everything is not informative and does not allow semantic differentiation. Similarly, we removed from the list those adjectives that modified fewer than 10 nouns both in the *animals* and in the *objects* class. That left 243 *animal* adjectives and 239 *object* adjectives (for a total of 360, with intersection 122).

Finally, we took into account the weights of the word graph edges, defined as the number of times the corresponding noun-adjective pair appeared in the original file of 32 million rows. For each noun we only kept the edges whose ranked weights added up to 75% of total weights for that given noun. The weights were then rescaled so that such sum would be approximately constant across nouns. This final pruning left 285 adjectives and 2,682 edges (1,324 originating from animals and 1,358 from objects). The results presented in the main text are those originated by the non-weighted version of this graph. Similar results were obtained with the weighted graph, and are reported in S.I. 3.1.

With the above word graph in place, we pre-trained two networks to learn half of the noun-adjective pairs from the graph. One of the networks learned more edges pertaining to animal nodes (becoming an animal expert and object novice), while the other learned more edges pertaining to object nodes (object expert, animal novice). Moreover, the amount of specialization was also varied to mimic different levels of specialization. The BIG ADO learning rule was then tested using a proximity threshold *θ* = 6.

In order to control for the general characteristics of this word graph independent of the actual distinction between the two noun classes, we created a set of equivalent random graphs preserving the overall degree distributions for both nouns and adjectives (Figure S1). Specifically, the random equivalent graphs were built stochastically from 100 “noun” and 285 “adjective” nodes by assigning edges to the noun-adjective pairs with probabilities corresponding to the degree distributions of Figure S1. Since the algorithm creates each edge independent of the others, the degree distribution of any individual random equivalent graph is slightly different, but their average distributions match those of the original graph.

***Figure S1.*** *Histogram distributions of noun and adjective degrees, binned by 5, in the word graph. For instance, there are 13 nouns that connect to a window of 33-37 adjectives (seventh bar of first panel) and 53 adjectives that receive connections from just 1 or 2 nouns (first bar of second panel).*

For BIG ADO testing, networks were pre-trained with expertise on an arbitrary subset of nodes of the random equivalent graph using the same parameters as in the real word graph.

**2.2: Watts-Strogatz networks**

Real-world networks can be simulated for many purposes with highly clustered, small-world graphs^[[2]](#endnote-2)^. Network clustering (or transitivity) measures how more likely two nodes are to connect with each other if they are both connected to the same other nodes^[[3]](#endnote-3)^. The small-world property consists of short minimal lengths between nodes on average (the mean shortest path length depends at most logarithmically on network size^[[4]](#endnote-4)^). Among several techniques to generate graphs with the above characteristics, the Watts-Strogatz (WS) method is well studied and offers a flexibility of parameters, making it the model of choice for many computational applications [main text: 19]. Briefly, the WS algorithm starts from an N-node ring with every node connected to its 2d nearest neighbors. Then, for all nodes, each edge to a clockwise node has a chance (R) of being removed and reconnected to a randomly chosen node. Typically, even low values of R (<0.1) result in small-world graphs with highly clustering index.

In order to generalize the results obtained with the word graph to more broadly applicable content, we created arbitrary reality-generating graphs based on a slight modification of the WS approach. Specifically, after producing a WS graph, a random direction is selected for each edge, while only a fraction of edges (10% in the results reported in the main text) is made bidirectional. Next, a subset (20%) of the nodes, along with all their incoming edges, is labeled as belonging to the agent’s area of expertise. The agent is then pre-trained (without BIG ADO filter) with a random set of edges of the graph, with the constraint that half of them must belong to the area of expertise, unless otherwise specified. As a result, the agent learns a sub-graph of the initial graph whose nodes in the area of expertise have higher average degree than those outside the agent’s expertise. In the “grandmother cell” implementation (Figures 3B and 3C in the main text), the BIG ADO threshold was set at 1.

**2.3: Associative Nets and optimal Bayesian learning models**

Many models have been proposed to store concepts and their associations with cell assemblies. These models differ in their details, complexity, and biological plausibility, but generally only explicitly represent excitatory neurons and finesse the inhibitory balance with algorithmic normalization such as k-winner-take-all^[[5]](#endnote-5)^.

One of the simplest such models is the Associative Net^[[6]](#endnote-6)^, which offers better biological relevance than the later spin glass formalism^[[7]](#endnote-7)^. Associative Nets work well for sparse patterns, that is, when the cell assembly sizes *S* (also called “activity”) is much smaller than the number of available neurons *N_n_*. If two concepts A and B are represented respectively by neurons a_1_, a_2_, …, a_s_ and b_1_, b_2_, …, b_s_, learning the association (A,B) follows the Hebbian principle of strengthening the synapses of co-activated neurons and weakening those between active and inactive neurons. Specifically, given an “incidence” matrix M in which rows and columns represent pre- and post-synaptic neurons, Associative Nets follow the following *homosynaptic* rule: when witnessing the co-occurrence of A and B, the entries in columns b_j_’s of all a_i_’s rows are increased by 1-r, while the remaining entries are decreasing by r (so as to keep zero-summing rows).

Retrieval in Associative Nets works as a dendritic sum: given a term A’ represented by neurons a’_1_, a’_2_, …, a’_s_ , the matrix-vector multiplication M x A’ is computed (adding up for each column all the entries in the rows corresponding to the a_i_’s). A threshold (for instance zero) can also be introduced to limit the entries contributing to the dendritic sum, whereas below-threshold values are interpreted as non-existing synapses. Those columns with dendritic sum above a certain value (Willshaw threshold) correspond to firing (post-synaptic) neurons. If enough neurons belonging to the same cell assembly B’ fire, concept B’ gets activated. Associative Nets are only efficient for very sparse patterns: the optimal activity is log_2_(N), amounting to cell assemblies of 15-20 neurons for systems with 10^6^ neurons^[[8]](#endnote-8)^. The maximum capacity decreases sharply as *r* increases, as catastrophic forgetting^[[9]](#endnote-9)^ becomes more and more likely.

To overcome the above limitations, a variant of this model was introduced, originally named “Zip Net”^[[10]](#endnote-10)^, which was later shown to achieve optimal Bayesian learning [main text: 22]. The Zip Net learning protocol is identical to that of Associative Nets, but the retrieval is different in the way the entries of the incidence matrix are interpreted. A key parameter of Zip Nets is the memory load *q*, defined as the fraction of 1-synapses or the probability that the weight of a given synapse is one. This parameter is kept fixed throughout the process, as follows. Given the original incidence matrix (updated with the linear learning rule), each column is treated separately, with only qN entries set to 1 in the connectivity matrix (those with the top values in the incidence matrix), and the rest set to zero. Therefore the connectivity matrix has exactly qN 1’s in each column. This is a form of homeostasis of the post-synaptic neuron, that tends to keep “open” only (and exactly) the strongest qN synapses it receives. The connectivity matrix is then used to perform the dendritic sum, with an appropriate threshold as before. The Zip Net model allows for larger storage capacity, larger assembly size, and more realistic biological interpretation^17^. In fact, Zip Nets do not work well with very small cell assembly sizes. Thus, their strengths can be considered complementary to those of Associative Nets.

Given the number of available neurons N_n_ and the cell assembly size S (two parameters of the model), we randomly choose one set of S neurons for each concept (node in the graph). In our implementation, neurons are points in a unit cube [0,1]^3^. The “center” of the assembly is selected randomly (uniformly), and neurons for that assembly are then drawn accordingly to a probability distribution that decreases with the Euclidean distance from the center. This specific algorithmic detail is irrelevant to the results of this work (assemblies could have been chosen arbitrarily), but is nonetheless reported for completeness.

Starting from an initial zero-valued square matrix labeled by the neurons, the Incidence Matrix (IM) is updated during pre-training with each association witnessed by the agent, according to the *homosynaptic* learning rule described above. At the end of pre-training, IM is a real-valued zero-sum matrix indicating which (row) neurons should be connected to which (column) neurons. It is therefore used in the following way. In the associative net model, the binary Synapse Matrix (SM) is created by setting to 1 all IM entries that are above a fixed threshold (a parameter of the algorithm), and to 0 the others. In the Zip Net model, a “Matrix Load” (ML) parameter (total number of synapses over possible number of synapses) is chosen, then the highest ML x N_n_ entries of each *column* in IM are set to 1 in SM, while the others are set to zero.

**2.4: BIG ADO implementation with cell assemblies**

The above cell assembly model implementations were used to connect the networks during pre-training. In the BIG ADO testing phase, if two neurons *a* and *b* are co-activated, they can only form a new synapse if they have an axonal-dendritic overlap. In the main text (Fig. 1A) of this paper, we expressed this condition with the proximity function, which in turn called into play the connectivity of third party neurons (*c*, *d*). In cell assembly models, however, the number of connected synapses is determined either by a threshold (Associative Nets) or as a normalized constant (Zip Net). In these models, therefore, the positive values of the Incidence Matrix, rather than the Synaptic Matrix, constitute the most faithful trace of witnessed co-occurrences, and should be used as proxies to determine optimal neuronal placement.

We thus define a new *vicinity* matrix VM as the Incidence Matrix in which negative entries are set to zero. This zeroing of negative numbers is necessary to avoid that their product would yield positive values, but can also be understood in anatomical terms: the chance of axonal-dendritic overlap between neurons *a* and *b* should increase based on the existence of appropriate *nearby* neurons *c* and *d*, but should not decrease based on the existence of far-away neurons *c* and *d*. Connectivity implies vicinity, but not vice-versa, since in Zip Nets the post-synaptic neuron may decide to shut down some of its synapses while remaining close to their axons.

In summary, we have:

VM = max (IM, 0)

Then we can apply the triple product to compute the proximity matrix:

PM = VM x VM^T^ x VM

Finally, axonal-dendritic overlaps are assigned to all pairs (a,b) whose entries in PM are in the top fraction, which is determined by another parameter, the proximity load (PL). Moreover, synaptically connected neurons as well as the pairs with the top (PL/2) entries in the IM are also considered to have axonal-dendritic overlaps.

**Part 3: Additional Results**

**3.1: Word association: graph analysis, weights, and cell assemblies**

The results of Figure 2B in the main text demonstrate that BIG learning works better for networks trained in *animals* than for networks trained in *objects*. This observation can be expanded by examining the graph properties of networks trained in the noun-adjective associations for the two classes of noun (Table S1).

|  | learnable edges | | mean proximity | | % edges with non-zero proximity | | mean proximity of non-zero edges | |
| --- | --- | --- | --- | --- | --- | --- | --- | --- |
|  | animals class | objects class | animals class | objects class | animals class | objects class | animals class | objects class |
| Animal Training | 997 | 1,254 | 3.80 | 0.22 | 62% | 12% | 6.15 | 1.83 |
| Object Training | 1,219 | 1,040 | 0.34 | 1.29 | 17% | 55% | 2.01 | 2.89 |

***Table S1****: Graph properties of networks specialized in noun-adjective associations for Animals or Objects.*

For an Animal-trained network, the mean proximity of a (not yet learned, i.e. available for learning) new association belonging to the animal class is much higher than that of an object class. Conversely, for an Object-trained network the mean proximity is higher for object associations. As with Figure 2B in the main text, these values tend to favor expert learning after Animal pre-training over expert learning after Object pre-training.

This result is not due to different average degrees of the respective nodes, which are in fact quite similar in the word association bipartite graph (1,324 edges from *animals* to adjectives and 1,358 from *objects* to adjectives). Instead, the reason is that the *animals* group is a more coherent group, in the sense that adjectives with high proximity to a noun tend to have high proximity with most of the nouns in the group, and vice-versa for those adjectives with low proximity.

Given two nouns N_1_, N_2_, we can compute the (Pearson) correlation between the two vectors V_1_ and V_2_ given by the proximity function of N_1_ and N_2_ with the 285 adjectives. Doing so for every pair of nouns we obtain a 100x100 symmetric matrix of Pearson’s cross-correlation coefficients. Its (i,j) entry is a measure of how the proximities values (with all adjectives) of noun i correlates to those of noun j. Nouns that share similar characteristics (adjectives) should have higher correlation coefficients. The average correlation between two (distinct) nouns belonging to the *animals* group amounts to C_a,a_ = 0.988 (s.d. 0.0008), the average correlation between two *objects* amounts to C_o,o_ = 0.945 (s.d. 0.0008), and the average cross correlation across members of the two groups amounts to C_a,o_ = 0.896 (s.d. 0.0009). Dividing the set of 100 nouns into two (randomly chosen) 50 element groups yields an average correlation of C_aa_ = C_oo_ = C_ao_ = 0.931 (s.d. 0.0009).

From these values it can be concluded that the internal cross-correlation for the *animal* class is very significantly (p<10^-12^) higher than expected by chance for a graph with this connectivity: animal nouns form a highly coherent group with respect to their associations with adjectives. In contrast, the *object* class’ internal correlation is only marginally higher (p<0.03) than expected by chance: *object* nouns are somewhat coherent, but far less so than the *animal* nouns. The correlation across group is very significantly (p<10^-100^) lower than expected by chance, confirming that the two noun classes are clearly separate.

This analysis reveals two distinct factors in the gating mechanism of learning by background information. One factor is linked to the individual history experienced by an agent, which results in a particular domain of expertise. A second factor depends on how internally informative a given domain of knowledge is. If the information learned about a topic is highly relevant to other aspects of that subject matter, a network pre-trained in that topic will more easily acquire new knowledge in the same subject area. The internal cross-correlation measured above is a mathematical characterization of this “intrinsic background information”. Thus, the BIG ADO mechanism can differentially facilitate learning based on (i) intrinsic background information given the same expertise, (ii) level of expertise given the same intrinsic background information or, in the most common and general case, (iii) a combination of the above.

In a separate set of simulations, networks were trained with a version of the word association bipartite graph in which edges were weighted based on the frequency of noun-adjective co-occurrence in the original corpus, as described in section 2.1 of this S.I. Background information gating clearly differentiates both expert from novice learning and *Animal* from *Object* learning in this case as well (Table S2), similar to the result obtained with the un-weighted model.

|  | learnable edges | | mean proximity | | % edges with non-zero proximity | | mean proximity of non-zero edges | |
| --- | --- | --- | --- | --- | --- | --- | --- | --- |
|  | animals class | objects class | animals class | objects class | animals class | objects class | animals class | objects class |
| Animal training | 1,065 | 1,260 | 3.23 | 0.24 | 52% | 9% | 6.22 | 2.60 |
| Object training | 1,241 | 1,082 | 0.47 | 1.43 | 11% | 35% | 4.08 | 4.14 |

***Table S2****: Graph properties of networks specialized in noun-adjective associations for Animals or Objects in the weighted bipartite graph.*

Moreover, we tested the BIG ADO learning rule on the word association bipartite graph also using cell assemblies. Both Willshaw’s Associative Nets and the optimal Bayesian learning Zip Nets yielded results consistent with the findings obtained with grandmother neuron simulations (Figure S2). Specifically, learning is systematically facilitated by pre-training in the same domain of expertise, and the effect is stronger for *Animals* than for *Objects*.

These additional results suggest that the word association learning results described in the main text are robust and not due to a specific model design or choice of model parameters. In the next section we extend the robustness analysis to the general Watts-Strogatz graphs.


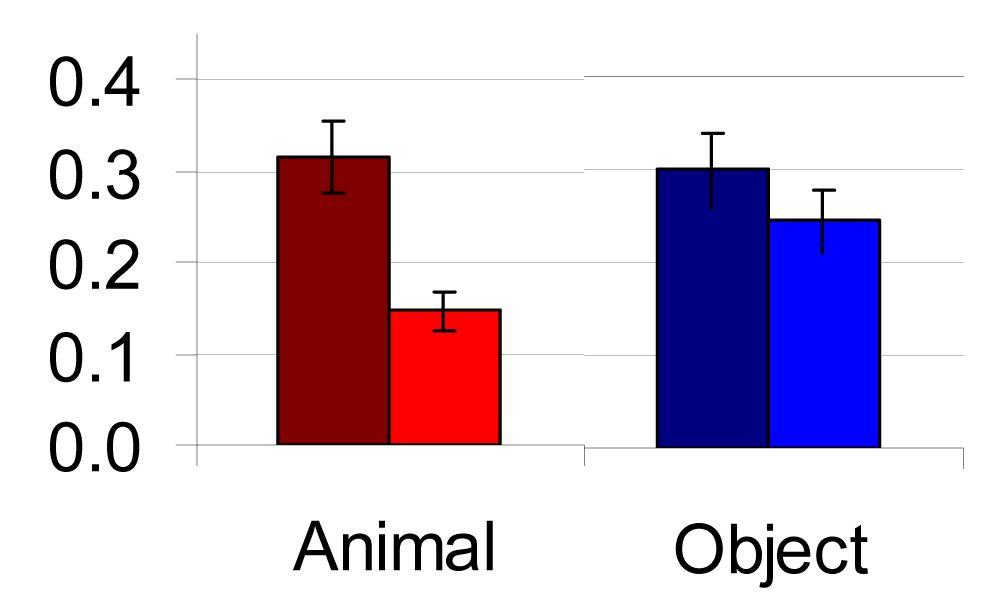

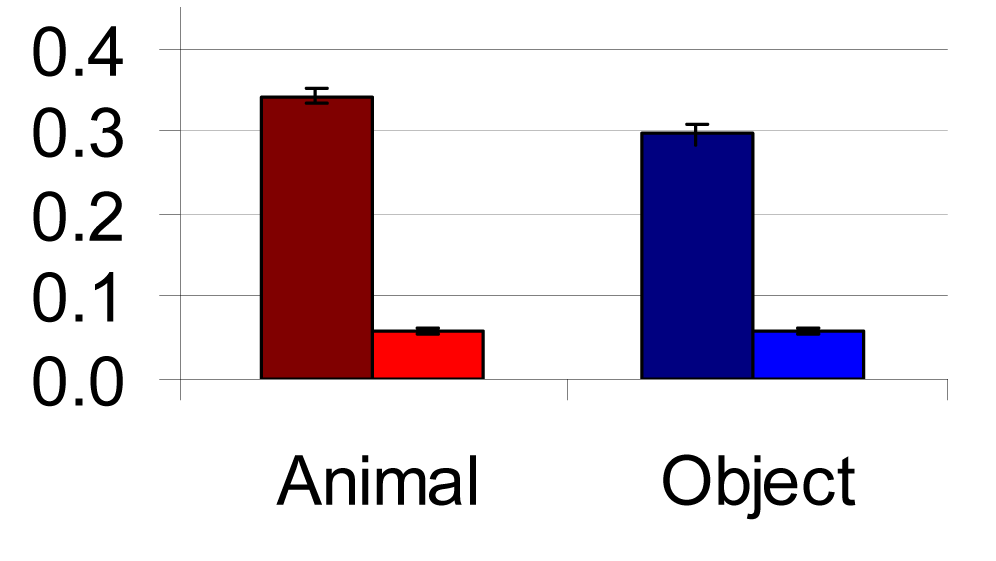


***Figure S2****: BIG ADO learning of word associations with Associative Nets (left panel: Number of cells = 250, assembly size = 5, Willshaw threshold = 0.75) and optimal Bayesian learning model (right: Number of cells = 400, assembly size = 10, Matrix Load = 0.2). Error bars are standard errors of the mean, based on 20 simulations.*

**3.2: From ADO to synapses: variations and robustness**

The relationship between proximity and the potential to form a synapse between two co-activated neurons constitutes an important aspect of the BIG ADO learning rule. In the simplest model, this relationship is defined as a fix threshold, corresponding to a model parameter *θ*, above which entries in the Proximity Matrix Π correspond to potential synapses. In order to test the effect of this threshold on the main conclusions of this study, we varied the parameter *θ* in the general Watts-Strogatz association graph models. Every value of *θ* leading to an amount of learning within a reasonable range between detectable and saturating levels confirmed a superior ability to form real associations as opposed to spurious ones, and within the domain of expertise as opposed to outside (Figure S3). Not surprisingly, the novice/expert ratio increases, indicating a reduced discriminating power of the BIG ADO learning rule, at low *θ* values conducive to indiscriminate learning. Interestingly, the real/spurious ratio remains fairly low even in these more extreme conditions.

0%

10%

20%

30%

40%

50%

60%

70%

80%

90%

5

10

15

20

**Threshold**

0%

10%

20%

30%

40%

50%

60%

70%

80%

90%

expert

novice

real

spurious

novice/expert

spurious/real

***Figure S3****: Proportion of expert, novice, real, and spurious learned associations (left axis, bars) and learning ratios (right axis, lines) as a function of the threshold parameter PT in WS graphs (500 nodes, of which 100 expert nodes; 5000 associations taught, of which 1200 expert; graph degree 30). Error bars are standard errors of the mean, based on 10 simulations.*

In addition to a sharp threshold cutoff, we also implemented a probabilistic version of the BIG ADO learning rule, with a sigmoid curve given by

PS(x) = (tan^-1^((x-PT)/g) + π/2)/π

where x is the proximity between two neurons and PS is their probability of forming a synapse upon co-activation. Here the parameter PT corresponds to the proximity value with a 50% probability, and PT+g, PT-g are the first and third quartile of the probability distribution. Thus, g is a parameter that specifies how steep (that is, how close to a sharp cut-off) the sigmoid is, with g=0 corresponding to the simple threshold model described above (Fig. S4).


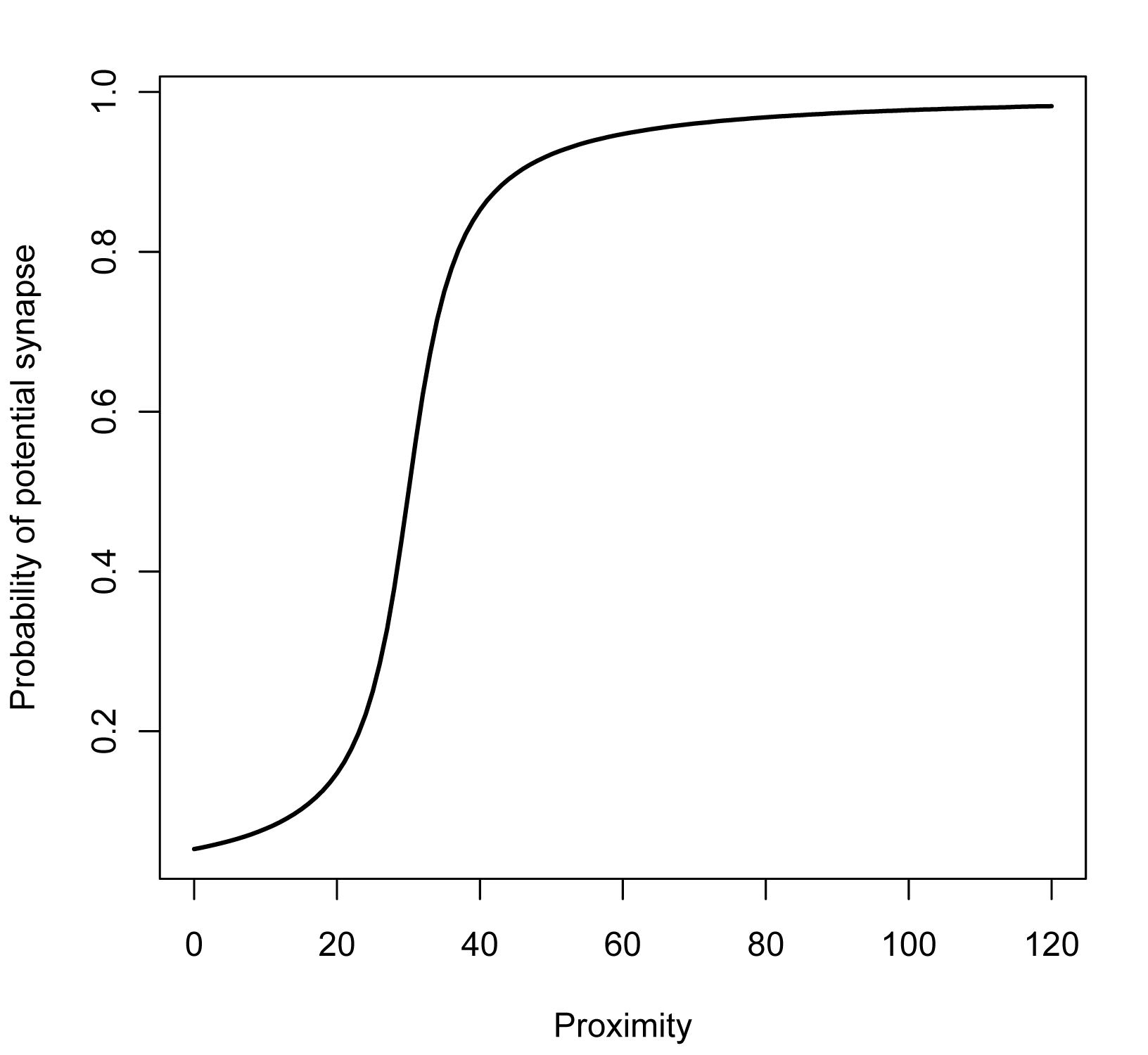


***Figure S4****: Example of sigmoid threshold to derive the probability of a potential synapse as a function of proximity.*

In our simulations (Figure S5), a decrease in sharpness is accompanied by an increase in PT; this is because a symmetric distribution (P(x)) is superimposed to the distribution of values in PM which is left-skewed, therefore keeping PT constant would result in an increase of all learning. In contrast, we tried to keep the average learning overall constant, in order to investigate the relative learning ability. We also included in the same set of experiments a simulation in which the probability of forming a synapse grows linearly with proximity up to a unitary value 1 for x=100, corresponding to a “flat” sigmoid.

0%

5%

10%

15%

20%

25%

30%

35%

15; 0

20; 2

30; 5

linear (100)

**PT; g**

0%

10%

20%

30%

40%

50%

60%

70%

expert

novice

real

spurious

novice/expert

spurious/real

***Figure S5:*** *Proportion of expert, novice, real, and spurious learned associations (left axis, bars) and learning ratios (right axis, lines) in the same WS graphs as in Fig. S5 for the sharp threshold (leftmost data, same as third group in Fig. S3), a steep sigmoid (PT=20, g=2), a shallow sigmoid (PT=30, g=5), and a linear probability (rightmost data). Error bars are standard errors of the mean, based on 20 simulations.*

Once again, every choice of function leading to an amount of learning within a reasonable range between detectable and saturating levels confirmed a superior ability to form real associations as opposed to spurious ones, and within the domain of expertise as opposed to outside (Figure S5). We conclude that background information-gated learning by axon-dendrite overlap is a robust computational mechanism over a variation of model design and parameter values.

**Supplementary Reference**

1. Barlow HB (1994) What is the computational goal of the neocortex? In C. Koch and J. Davis, Eds., Large Scale Neuronal Theories of the Brain. Cambridge, MA: MIT Press. [↑](#endnote-ref-1)
2. Boccaletti S, Latora V, Moreno Y, Chavez M, Hwang DU (2006) Complex networks: Structure and dynamics. *Phys. Reports* **424**: 175-308. [↑](#endnote-ref-2)
3. Wasserman S, Faust K (1994) *Social Networks Analysis.* Cambridge University Press, Cambridge UK. [↑](#endnote-ref-3)
4. Albert R, Barabási AL (2002) Statistical mechanics of complex networks. *Rev. Mod. Phys.* **74**: 47-97. [↑](#endnote-ref-4)
5. Maass W (2000) On the computational power of winner-take-all. *Neural Comput.* **12**: 2519-2535. [↑](#endnote-ref-5)
6. Dayan P, Willshaw DJ (1991) Optimising synaptic learning rules in linear associative memories. *Biol Cybern.* **65**: 253-265. [↑](#endnote-ref-6)
7. Hopfield JJ (1982) Neural networks and physical systems with emergent collective computational abilities. *Proc. Natl. Acad. Sci. U.S.A.* **79**: 2554-2558. [↑](#endnote-ref-7)
8. Graham B, Willshaw D (1997) Capacity and information efficiency of the associative net. *Network: Computation in Neural Systems* **8**: 35-54. [↑](#endnote-ref-8)
9. Robins A, McCallum S (1998) Catastrophic forgetting and the pseudorehearsal solution in Hopfield type networks. *Connection Science* **7**: 121-135. [↑](#endnote-ref-9)
10. Knoblauch A (2010) Zip nets: Efficient associative computation with binary synapses. Proceedings of the International Joint Conference on Neural Networks (IJCNN), pp 4271-4278. See also: Knoblauch A (2011) Zip nets: Neural associative networks with non-linear learning.

    HRI-EU (Honda Research Institute Europe GmbH) Report 09-03, v.1.0 (made publicly available on June 20, 2011). patent-de.com/20110120/EP2259214.html. [↑](#endnote-ref-10)
